# Genistein and Butein as Bioactive Polyphenols: Molecular Targets, Metabolic Regulation, and Mechanistic Insights

**DOI:** 10.3390/life16040615

**Published:** 2026-04-07

**Authors:** Moon-Kyun Cho, Yeji Lee, Ki Dam Kim, Min Hyuk Choi, Sukh Que Park, Sang-Han Lee, Hae-Seon Nam, Yoon-Jin Lee

**Affiliations:** 1 Division of Molecular Cancer Research, Soonchunhyang Medical Research Institute, Soonchunhyang University, Cheonan 31511, Republic of Korea; mkcho@schmc.ac.kr (M.-K.C.);; 2 Department of Dermatology, Soonchunhyang University Hospital, Seoul 04401, Republic of Korea; 3 Department of Biochemistry, College of Medicine, Soonchunhyang University, Cheonan 31511, Republic of Korea; 4 Department of Neurosurgery, Soonchunhyang University Hospital, Seoul 04401, Republic of Korea; 5 Department of Tropical Medicine, College of Medicine, Soonchunhyang University, Cheonan 31511, Republic of Korea

**Keywords:** genistein, butein, bioactive polyphenols, PI3K/Akt signaling, metabolic reprogramming, apoptosis, redox regulation, mechanistic insights

## Abstract

Bioactive polyphenols are increasingly recognized as modulators of multiple biological processes relevant to human health and disease. Among these compounds, genistein, a soy-derived isoflavone, and butein, a naturally occurring chalcone, have been investigated for their anticancer, anti-inflammatory, and metabolic regulatory activities, primarily in in vitro and preclinical experimental models. Despite their distinct chemical structures, available evidence indicates that genistein and butein can influence key molecular pathways involved in cell survival, energy metabolism, and programmed cell death. Experimental studies have shown that these compounds may modulate PI3K/Akt and MAPK/ERK signaling, alter glycolytic and mitochondrial metabolism, and induce apoptotic responses through caspase activation and poly(ADP-ribose) polymerase cleavage. This review provides a comprehensive overview of the chemical characteristics, bioavailability, and proposed molecular mechanisms of action of genistein and butein, with a particular focus on their potentially convergent roles in metabolic reprogramming and apoptotic signaling networks. In addition, we discuss the conceptual basis for combination approaches involving these compounds, emphasizing systems-level pathway modulation rather than definitive pharmacological synergy. Importantly, many of the reported biological effects have been observed under experimental conditions using concentrations that may exceed physiologically achievable concentrations, thereby limiting direct extrapolation to clinical settings. Furthermore, the current evidence base is constrained by limited in vivo validation and a lack of robust clinical data, particularly for butein. Future studies are required to better define pharmacokinetic properties, physiological relevance, and context-dependent biological effects, thereby providing a more rigorous framework for future evaluation of the translational potential of genistein and butein.

## 1. Introduction

Bioactive compounds from dietary and medicinal plants have garnered significant interest due to their diverse biological activities and potential roles in disease prevention and biological regulation [1,2,3]. Among these compounds, polyphenols form a major class of bioactive molecules that influence cellular signaling, energy metabolism, oxidative stress, and inflammatory responses—key processes involved in the onset and progression of chronic diseases such as cancer and metabolic disorders [4,5,6,7]. Unlike traditional pharmacological agents, which typically target a single molecular entity, polyphenols can simultaneously modulate multiple interconnected signaling and metabolic networks, facilitating coordinated and context-dependent regulation of complex cellular responses [8,9,10]. From a systems biology perspective, the capacity of dietary polyphenols to influence various signaling pathways, metabolic fluxes, and cell fate decisions aligns with the concept of Life, which underscores integrated biological regulation at molecular and cellular levels.

Among dietary polyphenols, genistein, a soy-derived isoflavone, has been widely investigated, primarily in in vitro and preclinical experimental models, for its reported anticancer, cardioprotective, and metabolic regulatory activities [11,12,13,14]. Due to its structural similarity to 17β-estradiol, genistein can interact with estrogen receptors and also affect growth factor signaling pathways. Notably, genistein has been reported to modulate the phosphoinositide 3-kinase (PI3K)/Akt and mitogen-activated protein kinase (MAPK)/extracellular signal-regulated kinase (ERK) pathways, which are crucial for cellular survival, proliferation, and adaptive stress responses across various physiological and pathological contexts [15,16,17,18]. In addition to its role in signal transduction, emerging evidence suggests that genistein may influence cellular energy metabolism by reducing glycolytic activity and regulating key metabolic enzymes, such as hexokinase 2 (HK2) and pyruvate dehydrogenase (PDH), thereby functionally linking growth signal modulation with metabolic stress responses [19,20,21,22]. Through this coordinated regulation of signaling and metabolism, genistein exemplifies a multi-target dietary polyphenol that modulates disease-relevant cellular states in experimental systems.

Butein, a naturally occurring chalcone found in various medicinal plants, has garnered attention for its reported anti-inflammatory, antioxidant, and antiproliferative activities, as demonstrated primarily in experimental models [23,24,25,26]. Unlike genistein, butein features an α,β-unsaturated carbonyl moiety that gives it electrophilic characteristics, allowing it to interact with redox-sensitive signaling proteins and transcriptional regulators [27,28,29]. Experimental studies indicate that butein can influence pro-survival and inflammatory signaling pathways, such as Akt, ERK, and nuclear factor-κB (NF-κB), while also disrupting mitochondrial function and intracellular redox balance [30,31,32,33,34]. This focus on redox and mitochondrial mechanisms contributes to growth suppression and apoptotic cell death across various disease models [35,36,37], establishing butein as a bioactive compound with a distinct mechanistic bias toward redox- and mitochondria-driven cellular regulation.

**Figure 1:**
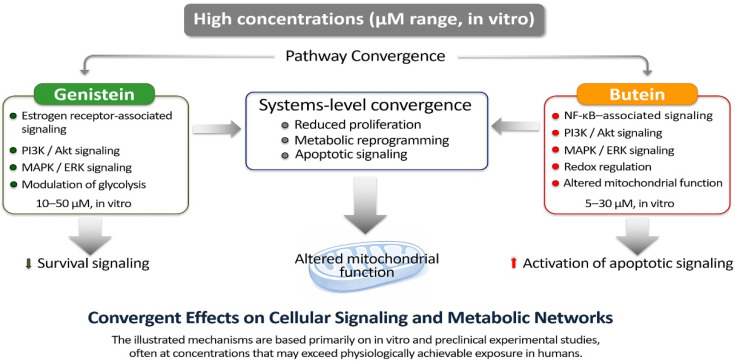
Integrated biological actions of genistein and butein on cellular signaling, metabolic regulation, redox balance, and apoptotic execution. Genistein primarily influences growth factor-associated signaling pathways and glycolytic metabolism, while butein mainly targets redox-sensitive signaling and mitochondrial function. Their combined effects on suppressing survival signaling, inducing metabolic stress, and activating apoptosis highlight a conceptual systems-level framework for combination strategies aimed at modulating disease-relevant cellular networks under experimental conditions. These interactions are supported primarily by preclinical experimental evidence obtained at micromolar concentrations commonly used in vitro. The schematic emphasizes context-dependent modulation of interconnected signaling and metabolic pathways rather than direct clinical or therapeutic implications.

Despite their different chemical structures, growing evidence suggests that genistein and butein may elicit convergent biological responses under experimental conditions [38,39,40,41]. The complementary chemical properties and regulatory actions of genistein and butein, which support these shared biological results, are detailed in Table 1 and visually represented in Figure 1. Both compounds have been reported to modulate survival-associated signaling, disrupt metabolic reprogramming, and facilitate apoptotic processes through caspase activation and poly(ADP-ribose) polymerase (PARP) cleavage [42,43,44,45,46]. Importantly, these shared outcomes arise from mechanistically complementary rather than redundant modes of action, reflecting coordinated modulation of interconnected regulatory networks rather than isolated pathway inhibition. Given the central roles of metabolic plasticity and apoptosis resistance in disease progression—not only in cancer but also in chronic metabolic and inflammatory disorders—this convergence provides a conceptual biological rationale for combination strategies utilizing bioactive polyphenols [47,48,49,50].

In recent years, combination approaches using bioactive compounds have emerged as a promising strategy to enhance efficacy while minimizing toxicity, particularly in complex diseases driven by multiple dysregulated signaling and metabolic networks [51,52,53,54]. Within this context, the pairing of genistein and butein is of particular interest, as it integrates complementary effects on growth factor-associated signaling, metabolic regulation, redox balance, and apoptotic pathways. By focusing on a limited number of mechanistically well-characterized compounds, the present review adopts a targeted, concept-driven approach, thereby avoiding the superficial treatment that often characterizes broad, compound-enumerative reviews [55,56,57,58].

Thus, this review offers a focused, mechanism-driven overview of genistein and butein as bioactive compounds relevant to medicine and health. We summarize their chemical properties and bioavailability, critically evaluate their molecular mechanisms of action, and highlight their combined effects on metabolic regulation and apoptotic signaling. Finally, we discuss the potential biological relevance and translational considerations of combination strategies and outline current limitations and future research directions to facilitate the rational and evidence-based evaluation of genistein- and butein-based approaches. Unlike previous reviews that primarily describe individual polyphenols, the present review emphasizes a comparative and systems-level perspective, highlighting convergent regulatory mechanisms linking survival signaling, metabolic adaptation, redox balance, and apoptotic susceptibility in the context of genistein–butein complementarity. To improve conceptual consistency and methodological transparency, the literature surveyed in this review was primarily identified through searches of major scientific databases, including PubMed, Web of Science, and Google Scholar. Emphasis was placed on peer-reviewed studies providing mechanistic insights into cellular signaling pathways, metabolic regulation, mitochondrial function, redox homeostasis, and apoptosis-related processes associated with genistein and butein.

**Table 1:** Systems-level characteristics of genistein and butein as bioactive polyphenols, including representative concentration ranges.

Feature	Genistein	Butein	Key References
Chemical class	Isoflavone	Chalcone	[11,12,13,14,23,24,25,26]
Core structural features	Diphenolic isoflavone scaffold; phytoestrogenic activity	α,β-unsaturated carbonyl moiety; electrophilic scaffold	[15,16,17,18,27,28,29]
Primary biological entry points	Estrogen receptor- and growth factor-associated signaling	Redox-sensitive signaling and transcriptional regulation	[15,16,17,18,30,31,32,33,34]
Dominant regulatory emphasis	Growth factor signaling; energy metabolism	Redox balance; mitochondrial integrity	[19,20,21,22,31,32,33,34]
Metabolic regulation	Reduced glycolytic flux; modulation of HK2 and PDH (typically 10–50 µM in vitro experimental models)	Mitochondrial dysfunction; redox imbalance (typically 5–30 µM in vitro experimental models)	[19,20,21,22,31,32,33,34]
Cell fate modulation	Metabolic stress-associated apoptosis (commonly 20–100 µM in vitro)	Oxidative stress-associated apoptosis (commonly 10–50 µM in vitro)	[35,36,37,42,43,44,45,46]
Apoptotic execution	Caspase activation; PARP cleavage (20–100 µM in experimental systems)	Caspase activation; PARP cleavage (10–50 µM in experimental systems)	[35,36,37,42,43,44,45,46]
Bioavailability considerations	Rapid metabolism; extensive conjugation	Limited systemic availability	[59,60,61,62,63,64,65,66]
Evidence base	Primarily in vitro studies with limited in vivo validation	Primarily in vitro studies with limited in vivo validation	[14,22,37]
Systems-level contribution	Signaling and glycolytic network modulation	Mitochondrial and redox network modulation	[47,48,49,50,51,52,53,54]

## 2. Chemical Characteristics and Bioavailability

### 2.1. Structural Features of Genistein

Genistein (4′,5,7-trihydroxyisoflavone) is a prominent isoflavone primarily found in soybeans and soy-derived products. It has been extensively researched as a bioactive dietary polyphenol, exhibiting a range of biological activities, including anticancer, cardioprotective, and metabolic regulatory effects primarily demonstrated in experimental models [59,60,61]. Structurally, genistein features a diphenolic isoflavone backbone with two aromatic rings linked by a heterocyclic pyran ring, distinguishing it from other flavonoid subclasses such as flavonols and flavanones [62]. This rigid and largely planar structure facilitates interactions with diverse protein targets, contributing to its relatively high biological activity observed under experimental conditions compared with less structurally constrained polyphenols.

The specific arrangement of hydroxyl groups at the 4′, 5, and 7 positions is crucial for determining genistein’s redox properties, binding affinity, and molecular interactions. These hydroxyl groups enhance its antioxidant capacity and facilitate hydrogen bonding with target proteins, thereby influencing enzymatic activity and transcriptional regulation [63]. Even minor variations in hydroxylation patterns among isoflavones can markedly alter biological potency, underscoring the structure–activity relationships underlying genistein’s diverse effects.

Due to its structural similarity to 17β-estradiol, genistein exhibits phytoestrogenic activity by binding to estrogen receptors (ERs), with a higher affinity for estrogen receptor β (ERβ) than for estrogen receptor α (ERα) [64,65]. ERβ plays key roles in growth regulation, cellular differentiation, and tumor-suppressive signaling in hormone-responsive tissues, providing a mechanistic basis for the context-dependent biological effects of genistein observed primarily in preclinical studies [66]. This receptor selectivity may contribute to differential cellular responses, where ERβ-dominant signaling is often associated with growth-inhibitory or differentiating effects, whereas ERα activation is more commonly linked to proliferative responses in certain normal tissues. Thus, the net biological outcome of genistein is context-dependent and influenced by the relative expression of ER subtypes.

In addition to its interactions with nuclear hormone receptors, genistein modulates multiple intracellular signaling pathways, including receptor tyrosine kinases and downstream cascades such as the phosphoinositide 3-kinase (PI3K)/Akt and mitogen-activated protein kinase (MAPK)/extracellular signal-regulated kinase (ERK) pathways.

Notably, genistein has been reported to inhibit tyrosine kinase activity with an IC50 of approximately 50 μM, whereas physiologically achievable concentrations in vivo are typically in the pico- to low nanomolar range, highlighting a discrepancy between experimental and physiological conditions that should be considered when interpreting biological relevance [59,60,61,62]. These pathways collectively regulate cellular survival, proliferation, and metabolic homeostasis [67,68,69].

At the molecular level, the hydroxylated aromatic rings of genistein enable hydrogen bonding and π–π stacking interactions with kinases, transcription factors, and regulatory proteins, facilitating coordinated modulation of interconnected signaling and metabolic networks. Molecular rigidity further contributes to this process by stabilizing ligand–protein interactions and enabling sustained engagement with multiple signaling nodes, thereby influencing both metabolic and survival pathways. Together, these structural features support the pleiotropic biological actions of genistein, reinforcing its classification as a multi-target bioactive compound relevant to systems-level and combination-based intervention strategies. The key structural features of genistein and their functional implications are summarized in Table 2.

### 2.2. Structural Features of Butein

Butein (3,4,2′,4′-tetrahydroxychalcone), a member of the chalcone subclass of flavonoids, is found in various medicinal plants, including Rhus verniciflua and Dalbergia odorifera, which have been used in traditional medicine for centuries [71,72,73]. These plants have been traditionally associated with anti-inflammatory, antioxidant, antimicrobial, and anticancer properties based primarily on experimental and ethnopharmacological observations. Unlike genistein, which possesses a closed heterocyclic ring system, butein features an open-chain flavonoid structure consisting of two aromatic rings linked by an α,β-unsaturated carbonyl group [74]. This open-chain configuration confers greater conformational flexibility and electrophilic reactivity, resulting in physicochemical and biological properties distinct from those of other dietary polyphenols.

A defining structural characteristic of butein is its α,β-unsaturated carbonyl group, which functions as a Michael acceptor. This feature enables covalent or reversible interactions with nucleophilic residues, particularly cysteine thiols, in redox-sensitive signaling proteins [75,76]. Through such interactions, butein can modulate the activity of transcription factors and signaling regulators involved in inflammation, oxidative stress responses, and cell survival, including nuclear factor-κB (NF-κB) and components of the Akt and ERK pathways as demonstrated primarily in biochemical and cellular studies [77,78,79]. These electrophile-driven mechanisms distinguish butein from polyphenols that act predominantly through receptor-mediated or non-covalent interactions. Notably, this electrophilic reactivity allows butein to interact with electron-rich nucleophilic sites rather than directly acting as an electron donor, thereby contributing to its unique modulation of redox-sensitive signaling pathways.

In addition, the multiple hydroxyl groups on butein’s aromatic rings enhance its antioxidant capacity and influence interactions with cellular membranes, enzymes, and redox-regulating proteins. These hydroxyl groups participate in hydrogen bonding and redox reactions, thereby contributing to the regulation of intracellular redox homeostasis observed in experimental models [80]. Importantly, the coexistence of electrophilic reactivity and antioxidant potential allows butein to exert context-dependent effects on oxidative stress signaling, rather than functioning solely as a radical scavenger.

The chalcone structure of butein has been consistently associated with growth-inhibitory and pro-apoptotic responses primarily in in vitro experimental models, including cancer and inflammatory conditions [81,82]. Furthermore, butein has been reported to inhibit tyrosine kinase activity at concentrations exceeding approximately 50 μM, indicating that its direct kinase-targeting effects occur predominantly within the micromolar range under experimental conditions.

These effects are frequently linked to modulation of redox balance, mitochondrial integrity, and apoptotic signaling. Accordingly, butein represents a class of bioactive compounds whose chemical architecture enables mechanistic investigation of redox-sensitive and mitochondrial regulatory pathways.

**Figure 2:**
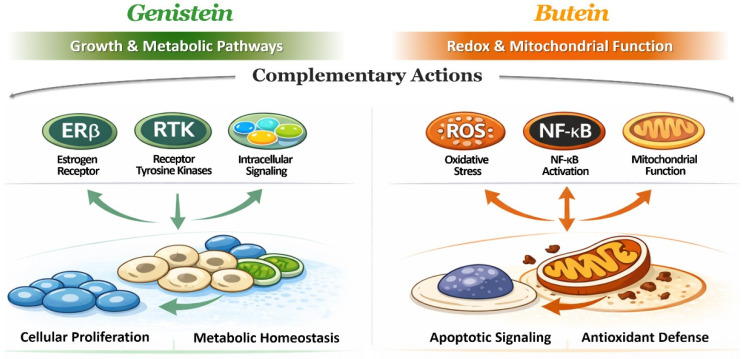
Functional complementarity of genistein and butein in cellular regulatory networks. Genistein predominantly modulates growth factor-associated signaling and metabolic pathways through receptor-dependent and kinase-targeting mechanisms, thereby influencing cellular proliferation and energy metabolism in experimental models. In contrast, butein preferentially targets redox-sensitive signaling and mitochondrial function via its electrophilic chalcone scaffold. Together, these complementary regulatory properties enable coordinated modulation of interconnected signaling, metabolic, and apoptotic networks under experimental conditions relevant to disease-associated cellular regulation. These interactions are primarily supported by preclinical experimental evidence derived from in vitro studies and reflect context-dependent modulation of cellular signaling and metabolic pathways rather than direct clinical or therapeutic effects.

In summary, the distinctive chemical properties of butein complement those of genistein, establishing a structural basis for their convergent yet non-redundant biological effects. While genistein primarily modulates growth factor-associated signaling and metabolic regulation, butein preferentially targets redox-sensitive signaling and mitochondrial pathways. This structural and functional complementarity provides a coherent mechanistic rationale for considering genistein and butein as a paired combination strategy in experimental research contexts, as illustrated in Figure 2. The primary structural features of butein and their functional implications are summarized in Table 3.

In addition, reported butein concentrations in plant sources vary widely depending on species, plant part, and extraction conditions, generally ranging from trace levels to measurable amounts in medicinal plant materials with variability across experimental analytical methods.

### 2.3. Absorption, Metabolism, and Bioavailability

Genistein and butein, despite their well-documented biological activities, share a common pharmacokinetic limitation typical of dietary polyphenols: restricted oral bioavailability [83,84]. This limitation is often viewed as a barrier to translational application. However, accumulating evidence suggests that these pharmacokinetic properties may influence the physiological context in which polyphenols exert biological effects, rather than constituting an intrinsic drawback.

Genistein is primarily present in soy-derived foods as glycosylated conjugates, such as genistin, which exhibit limited intestinal permeability in their intact form [85]. Notably, conjugated isoflavones generally exhibit lower intrinsic antioxidant and biological activity compared with their corresponding aglycone forms, emphasizing the importance of metabolic conversion for functional activity. Absorption depends on enzymatic hydrolysis by intestinal β-glucosidases of both host and microbial origin, releasing the aglycone prior to uptake. Importantly, the gut microbiota plays a critical role in this process, as microbial metabolism can generate bioactive metabolites that differ from the ingested compounds in both structure and function. In some cases, these microbiota-derived metabolites may represent the primary active forms in vivo rather than the parent compound.

Following absorption, genistein undergoes extensive phase II metabolism—mainly glucuronidation and sulfation—in enterocytes and hepatocytes, resulting in low circulating levels of free aglycone despite relatively high plasma concentrations of conjugated metabolites [86,87,88]. Therefore, the biologically relevant forms present in circulation and tissues are predominantly conjugated metabolites, and the functional properties observed in vivo may differ substantially from those of the native aglycone used in many in vitro studies. This metabolic profile highlights a key translational consideration: systemic exposure largely reflects metabolites rather than the parent compound, necessitating cautious interpretation of in vitro studies employing supraphysiological concentrations of aglycone genistein, which are typically in the micromolar range, whereas circulating concentrations in vivo are generally in the pico- to nanomolar range.

Butein exhibits a similarly limited pharmacokinetic profile. After oral administration, it is rapidly metabolized and conjugated, leading to low systemic exposure and a short plasma half-life [89,90]. While its electrophilic chalcone scaffold and hydroxyl groups confer potent redox-reactive and signaling-modulatory properties, these same features also render butein susceptible to rapid metabolic clearance, illustrating a broader principle in polyphenol biology whereby bioactivity and metabolic instability are intrinsically linked. As with genistein, the biologically active species in vivo may not correspond to the native compound but rather to its circulating or locally generated metabolites, further complicating direct extrapolation from in vitro findings.

Importantly, limited bioavailability should not be viewed solely as a translational limitation. Increasing evidence indicates that polyphenol metabolites, including glucuronidated and sulfated derivatives, may retain biological activity or exert distinct context-dependent effects influenced by tissue exposure, cellular uptake, and local enzymatic deconjugation [91]. Conjugated metabolites may therefore contribute to the observed biological effects either directly or following local deconjugation within target tissues, suggesting that metabolite-mediated activity may partially explain differences between in vitro potency and physiologically achievable concentrations. Tissues directly exposed to high concentrations of polyphenols and their metabolites, such as the intestinal epithelium and liver, may therefore represent primary sites of action for metabolic regulation, inflammation, and redox homeostasis.

From a limitation–opportunity perspective, combination-based strategies represent a physiologically aligned approach. Using structurally and mechanistically complementary compounds such as genistein and butein enables coordinated modulation of interconnected signaling and metabolic networks in experimental and theoretical frameworks, potentially reducing reliance on high systemic exposure to any single compound [92,93].

**Figure 3:**
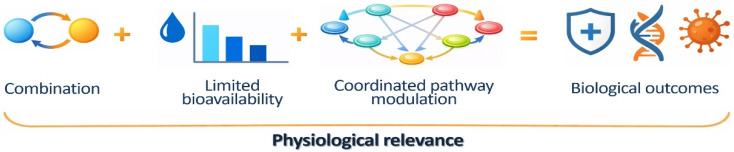
Pharmacokinetic context underlying combination-based interpretations of genistein and butein. Schematic overview of intestinal absorption and metabolic transformation of genistein and butein. Extensive phase II metabolism limits systemic exposure to the parent compounds, resulting predominantly in conjugated circulating forms and establishing a physiological framework for context-dependent multi-target modulation at tissue and cellular levels primarily observed in experimental studies. This schematic illustrates pharmacokinetic constraints that may influence the interpretation of in vitro findings obtained at micromolar concentrations relative to physiologically achievable exposure ranges.

In parallel, advances in formulation science—including nano-delivery systems, encapsulation technologies, and optimization of dietary matrices—have demonstrated potential to improve the stability, absorption, and tissue distribution of polyphenols under experimental conditions without altering their fundamental biological properties [94]. Collectively, these insights support a reframing of bioavailability constraints as defining parameters that shape physiological modes of action, rather than disqualifying limitations. In this context, genistein- and butein-based approaches are particularly well suited for integrative strategies requiring further experimental validation for health- and disease-related biological modulation. The key limitation–opportunity relationships affecting their pharmacokinetic behavior are summarized in Table 4, and the conceptual interplay between absorption and metabolism relevant to combination-based interpretations is illustrated in Figure 3.

## 3. Molecular Mechanisms of Genistein

### 3.1. Regulation of Survival Signaling Pathways

Genistein has been well documented as a modulator of key survival signaling pathways that are frequently dysregulated in cancer and other chronic diseases [95,96,97]. Notably, the phosphoinositide 3-kinase (PI3K)/Akt and mitogen-activated protein kinase (MAPK)/extracellular signal-regulated kinase (ERK) pathways play central roles in integrating extracellular growth signals with intracellular processes governing proliferation, metabolic adaptation, and resistance to cell death [98,99,100]. Persistent activation of these pathways is a hallmark of many malignancies, contributing to uncontrolled cellular growth, metabolic reprogramming, and resistance to therapy. Unless otherwise specified, the majority of mechanistic insights discussed in this section are derived primarily from cell-based experimental systems, with comparatively limited validation in animal models and human studies.

Importantly, the majority of mechanistic insights described in this context are derived from in vitro studies, where genistein is typically applied at micromolar concentrations (commonly ~10–100 μM). These exposure levels may exceed plasma concentrations achievable through dietary intake, which are generally in the low micromolar or sub-micromolar range. Therefore, interpretation of these findings should consider the potential gap between experimental conditions and physiological exposure when evaluating biological relevance.

Numerous experimental studies have demonstrated that genistein reduces the phosphorylation and activation of Akt and ERK, thereby attenuating the transmission of downstream survival signals [101,102,103]. These inhibitory effects are most consistently observed at concentrations ≥ 10 μM in vitro cancer cell models, where suppression of Akt activity has been linked to reduced signaling through key downstream effectors, including the mammalian target of rapamycin (mTOR) and glycogen synthase kinase-3β (GSK-3β). These effects result in impaired protein synthesis, altered metabolic regulation, and inhibition of cell cycle progression in experimental systems [104,105]. Concurrently, genistein-mediated modulation of ERK signaling disrupts transcriptional programs that promote oncogenic proliferation, survival, and adaptive stress responses in preclinical models [106].

Importantly, the effects of genistein on PI3K/Akt and MAPK/ERK signaling should not be interpreted as uniform pathway inhibition. Increasing evidence indicates that genistein modulates the overall signaling “tone” of these pathways in a context-dependent manner. At lower concentrations (≤1–5 μM), which are closer to physiological exposure levels, genistein may exert more subtle regulatory effects rather than robust pathway suppression. In contrast, in transformed or metabolically stressed cells, in which survival signaling is constitutively upregulated, higher concentrations tend to induce more pronounced inhibitory responses under experimental conditions [107].

Beyond its direct effects on survival signaling, the PI3K/Akt and ERK pathways are tightly coupled to cellular metabolic regulation, linking growth factor signaling to glycolytic flux, mitochondrial activity, and biosynthetic capacity. Accordingly, attenuation of these pathways at pharmacologically relevant concentrations contributes to coordinated disruption of metabolic processes that support sustained proliferation and stress adaptation in experimental models. By attenuating these pathways, genistein indirectly disrupts the metabolic reprogramming that supports sustained proliferation and survival under stress conditions. This coupling of signaling attenuation and metabolic disruption provides a mechanistic basis for the coordinated suppression of survival signaling and induction of metabolic stress observed in genistein-treated cells, positioning genistein as a regulator of interconnected survival and metabolic networks rather than a single-pathway inhibitor.

From a systems biology perspective, the context-dependent modulation of PI3K/Akt and ERK signaling by genistein likely contributes to its favorable biological profile observed in preclinical research. This apparent functional selectivity may, in part, reflect the differential sensitivity of cancer cells to signaling perturbations at higher concentrations, whereas basal signaling in non-malignant cells exposed to lower physiological levels remains comparatively preserved. By dampening aberrantly amplified survival signals while preserving basal signaling required for normal cellular function, genistein achieves a degree of functional selectivity that is particularly relevant for chronic disease modulation within experimental frameworks. These properties support its consideration as a bioactive compound suitable for integrative and combination-based strategies aimed at restoring balanced cellular regulation rather than enforcing complete pathway suppression.

### 3.2. Effects on Cellular Metabolism

In addition to its role in inhibiting survival signaling, genistein has emerged as a key regulator of cellular metabolism, which is increasingly recognized as being tightly linked to oncogenic signaling and disease progression [108,109]. Rapidly proliferating cancer cells often rely on enhanced glycolytic activity and altered mitochondrial function to meet biosynthetic demands and maintain redox balance, a phenomenon commonly referred to as metabolic reprogramming [110]. Genistein disrupts these adaptive metabolic processes at multiple regulatory levels, thereby linking modulation of growth-associated signaling to metabolic vulnerability in experimental models. Importantly, these metabolic effects have been predominantly characterized in vitro at micromolar concentrations of genistein (typically ~10–100 μM), which may exceed physiologically achievable plasma levels, highlighting the need for careful interpretation in a translational context.

Experimental studies indicate that genistein reduces glucose uptake and suppresses glycolytic flux, resulting in decreased lactate production and lower intracellular ATP levels [111,112,113]. These metabolic alterations are associated with the down-regulation or functional inhibition of key enzymes involved in glucose utilization and energy production, including hexokinase 2 (HK2), which catalyzes the first committed step of glycolysis, and pyruvate dehydrogenase (PDH), a central regulator controlling the entry of glycolytic intermediates into the tricarboxylic acid cycle [114,115,116]. These effects are most consistently observed at concentrations ≥ 10–50 μM, where significant reductions in glycolytic throughput and ATP production are reported, and by concurrently impairing glycolytic throughput and mitochondrial substrate utilization, genistein compromises metabolic flexibility and imposes energetic stress on cancer cells that depend on high metabolic plasticity for survival under experimental conditions.

The metabolic actions of genistein are closely interconnected with its modulation of growth factor-associated signaling pathways rather than representing isolated metabolic events. The PI3K/Akt axis plays a pivotal role in coordinating nutrient uptake, glycolysis, mitochondrial activity, and anabolic biosynthesis [117]. Attenuation of Akt signaling by genistein therefore leads to a coordinated suppression of survival and metabolic pathways, which can exacerbate metabolic stress in cells driven by oncogenic signaling in preclinical experimental systems [118].

Such coordinated effects are particularly evident at pharmacologically relevant concentrations, whereas at lower, physiologically relevant levels (≤1–5 μM), genistein may exert more subtle modulatory influences rather than inducing pronounced metabolic suppression. This integrated regulation helps explain the preferential sensitivity of transformed or metabolically stressed cells to genistein, while normal cells with lower metabolic demands remain comparatively less affected in experimental observations.

Beyond its effects on glycolysis, increasing evidence suggests that genistein also influences broader aspects of cellular metabolic homeostasis, including mitochondrial function, redox balance, and energy-sensing pathways. Reduced ATP availability following genistein treatment may activate cellular energy stress responses, such as AMP-activated protein kinase (AMPK) signaling, thereby further constraining biosynthetic capacity and cell growth in cell-based models. These responses are closely linked to the extent of ATP depletion, which is typically more pronounced at higher genistein concentrations, further emphasizing the dose-dependent nature of its metabolic effects. Through these interconnected mechanisms, genistein acts not merely as an inhibitor of individual metabolic enzymes but as a modulator of global metabolic network behavior.

From a systems biology perspective, genistein’s capacity to simultaneously attenuate growth factor signaling and metabolic flux positions it as a bioactive compound capable of targeting disease-associated metabolic vulnerabilities in experimental research contexts. Rather than inducing complete metabolic shutdown, genistein shifts cellular metabolic states toward conditions that are incompatible with sustained proliferation and survival under stress in preclinical models. However, the extent of this metabolic reprogramming is likely dependent on exposure level and duration, and may differ between pharmacological and physiological conditions. These characteristics underscore the potential of genistein as a component of integrative and combination-based strategies aimed at restoring balanced metabolic regulation in complex disease contexts requiring further experimental validation.

### 3.3. Induction of Cell Cycle Arrest and Apoptosis

Genistein is well recognized for its ability to induce cell cycle arrest and promote apoptotic cell death across a wide range of experimental models. However, the specific phase of cell cycle arrest and the dominant apoptotic mechanisms vary depending on cell type, concentration, and duration of exposure [119,120,121]. Notably, most of these effects have been characterized in vitro using micromolar concentrations of genistein (typically ~10–100 μM), which may exceed physiologically achievable plasma levels, underscoring the importance of dose-dependent interpretation when evaluating biological relevance.

Cell cycle arrest induced by genistein has been reported at the G0/G1, S, or G2/M phases and is frequently accompanied by coordinated alterations in cyclins, cyclin-dependent kinases, and endogenous cell cycle inhibitors such as p21Cip1 and p27Kip1 [122,123]. These regulatory effects are generally observed at concentrations ≥ 10–20 μM, whereas lower concentrations may exert more modest or context-dependent influences on cell cycle progression in experimental systems. This heterogeneity underscores the context-dependent nature of genistein’s regulatory actions and its capacity to interfere with cell cycle progression at multiple regulatory checkpoints.

In parallel with its effects on cell cycle regulation, genistein activates apoptotic pathways through both intrinsic and extrinsic mechanisms. Activation of the intrinsic mitochondrial pathway is supported by evidence of mitochondrial membrane potential loss, cytochrome c release, and subsequent activation of initiator caspase-9, followed by effector caspases [124,125,126]. In certain cellular contexts, genistein also modulates death receptor-associated signaling, thereby engaging extrinsic apoptotic pathways and further amplifying apoptotic execution. A consistent molecular hallmark across these models is the activation of caspase-3 and the cleavage of poly(ADP-ribose) polymerase (PARP), indicating irreversible commitment to programmed cell death in preclinical studies [127,128,129]. Importantly, these apoptotic events are strongly dose-dependent and are most prominently observed at concentrations ≥ 20–50 μM, suggesting that robust apoptosis induction may primarily reflect pharmacological exposure conditions.

**Figure 4:**
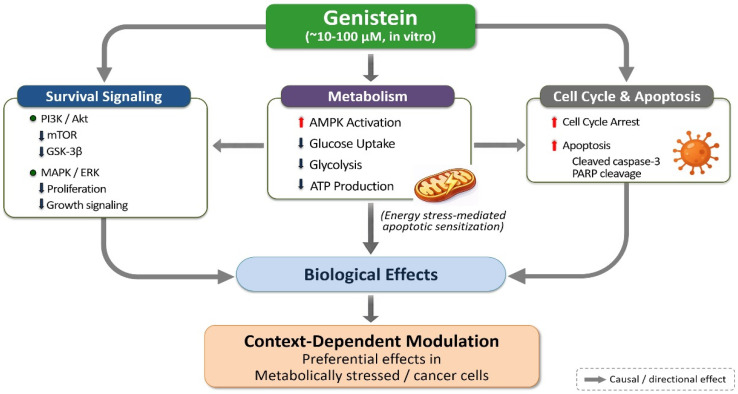
Integrated regulation of cell cycle arrest, metabolic stress, and apoptosis by genistein. Genistein attenuates PI3K/Akt and MAPK/ERK survival signaling, leading to coordinated suppression of cell cycle progression and metabolic activity in experimental models. Reduced glycolytic activity and mitochondrial energy production impose metabolic stress, which may contribute to increased susceptibility to apoptotic signaling. These convergent regulatory effects are associated with cell cycle arrest and activation of apoptotic pathways, including caspase activation and PARP cleavage, illustrating a systems-level mechanism by which genistein modulates cell fate under experimental conditions relevant to disease-associated cellular models. These mechanistic relationships are primarily supported by preclinical studies conducted at micromolar concentrations commonly used in vitro and reflect context-dependent modulation of interconnected signaling and metabolic pathways.

Importantly, genistein-induced apoptosis does not occur in isolation but is closely coupled to upstream suppression of survival signaling and disruption of metabolic homeostasis. Attenuation of PI3K/Akt and ERK signaling diminishes pro-survival cues, while impairment of glycolytic flux and mitochondrial energy production imposes metabolic stress. Together, these converging effects lower the apoptotic threshold and sensitize cells to programmed cell death. At lower, physiologically achievable concentrations (≤1–5 μM), these effects may be attenuated or require prolonged exposure to achieve comparable outcomes, highlighting the importance of exposure duration and cellular context in experimental observations. This integrated sequence—suppression of survival signaling, induction of metabolic stress, and execution of apoptosis—is conceptually summarized in Figure 4.

From a systems biology perspective, genistein’s capacity to concurrently influence cell cycle progression, metabolic state, and apoptotic machinery positions it as a regulator of cell fate rather than a nonspecific cytotoxic agent. By reshaping regulatory networks governing proliferation and survival, genistein promotes growth arrest and apoptosis in cells dependent on pathological signaling states in experimental models. This apparent selectivity may, in part, reflect the differential sensitivity of transformed cells to metabolic and signaling perturbations at higher genistein concentrations, whereas normal cells exposed to lower physiological levels remain comparatively less affected. Such coordinated, multi-layered regulation represents a defining feature of bioactive polyphenols and provides a mechanistic explanation for the favorable selectivity of genistein observed across diverse experimental systems.

Collectively, these findings support the consideration of genistein as a bioactive compound relevant to combination-based experimental strategies aimed at coordinated modulation of survival signaling, metabolic regulation, and apoptotic susceptibility. However, it should be noted that many of the described effects are observed at micromolar concentrations in vitro, and their translational relevance may depend on achievable systemic or tissue-specific exposure levels. Table 5 provides a systems-level summary of the regulatory layers influenced by genistein and their functional consequences, emphasizing the integrated modulation of survival signaling, metabolic stress, and apoptotic execution that underpins its biological activity.

## 4. Molecular Mechanisms of Butein

### 4.1. Modulation of Redox Homeostasis and Inflammatory Signaling

Butein is a bioactive chalcone known for its significant regulatory effects on redox homeostasis and inflammatory signaling pathways, both of which are crucial in the initiation, progression, and adaptation to disease-related stress in experimental models [130,131,132]. A key chemical characteristic of butein is its α,β-unsaturated carbonyl group, which confers electrophilic properties that facilitate interactions with nucleophilic residues, particularly cysteine thiols, in redox-sensitive proteins [133]. Through these interactions, butein modulates signaling pathways governing oxidative stress responses, inflammation, and cell survival primarily in cell-based systems. Compared with genistein, the mechanistic literature describing butein remains relatively limited and is derived predominantly from cell-based experimental systems, with comparatively fewer in vivo investigations and minimal translational or clinical validation currently available. Importantly, the majority of these mechanistic insights are derived from in vitro studies, where butein is typically applied at micromolar concentrations (commonly ~10–50 μM), which may exceed physiologically achievable plasma or tissue levels. Therefore, interpretation of these findings should consider the potential gap between experimental exposure and in vivo conditions when evaluating biological relevance.

Numerous studies have shown that butein inhibits nuclear factor-κB (NF-κB) signaling by blocking upstream kinases and preventing the nuclear translocation of NF-κB subunits, leading to decreased transcription of pro-inflammatory and pro-survival genes [134,135,136,137]. These inhibitory effects are most consistently observed at concentrations ≥ 10 μM in vitro, suggesting that robust suppression of NF-κB signaling may primarily reflect pharmacological exposure conditions in experimental studies.

Additionally, butein influences antioxidant defense systems by modulating redox-sensitive transcription factors and signaling intermediates, thereby altering intracellular levels of reactive oxygen species (ROS) in a context-dependent manner [138]. At higher concentrations, butein can promote ROS accumulation and oxidative stress in transformed or metabolically stressed cells, whereas at lower concentrations (≤1–5 μM), it may exert more subtle regulatory or antioxidant-like effects depending on cellular context in experimental observations. Importantly, this redox modulation is not merely a simple antioxidant effect; rather, butein can induce oxidative stress in transformed or metabolically stressed cells while maintaining redox balance in non-malignant tissues under controlled experimental conditions.

Redox and inflammatory signaling are closely linked to survival pathways such as PI3K/Akt and MAPK/ERK. Disruption of redox homeostasis by butein has been shown to indirectly attenuate these survival pathways, sensitizing cells to growth inhibition and apoptosis in preclinical models [139,140,141]. This coordinated regulation is strongly influenced by exposure level, with more pronounced inhibition of survival signaling observed at micromolar concentrations commonly used in vitro. Through this coordinated regulation, butein functions as a modulator of cellular stress responses rather than a single-pathway inhibitor. From a systems biology perspective, such dose- and context-dependent modulation may contribute to the apparent functional selectivity of butein, particularly in cells experiencing oxidative or metabolic stress in experimental systems.

### 4.2. Effects on Mitochondrial Function and Cellular Metabolism

In addition to its effects on redox and inflammatory signaling, butein significantly impacts mitochondrial function and cellular energy metabolism in experimental systems [142,143]. Mitochondria act as central hubs integrating metabolic status, redox balance, and cell fate decisions, and mitochondrial dysfunction is increasingly recognized as a therapeutic target in cancer and other chronic metabolic disorders [144]. Importantly, these mitochondrial effects have been primarily characterized in vitro using micromolar concentrations of butein (typically ~10–50 μM), which may exceed physiologically achievable plasma or tissue levels. Therefore, careful interpretation is required when extrapolating these findings to in vivo contexts and physiological exposure conditions.

Experimental evidence indicates that butein disrupts mitochondrial membrane potential, impairs oxidative phosphorylation, and reduces intracellular ATP levels in cell-based models [145,146,147].

These effects are most consistently observed at concentrations ≥ 10–20 μM, where significant impairment of mitochondrial function and ATP production has been reported. These changes are often accompanied by alterations in mitochondrial reactive oxygen species (ROS) production and modulation of enzymes involved in energy generation. At higher concentrations, butein tends to enhance mitochondrial ROS accumulation and induce oxidative stress, whereas at lower concentrations (≤1–5 μM), its effects on mitochondrial function may be more subtle and context-dependent in experimental observations. By compromising mitochondrial efficiency, butein induces metabolic stress that limits the bioenergetic capacity of diseased cells and constrains their ability to adapt to environmental or therapeutic challenges under experimental conditions.

**Figure 5:**
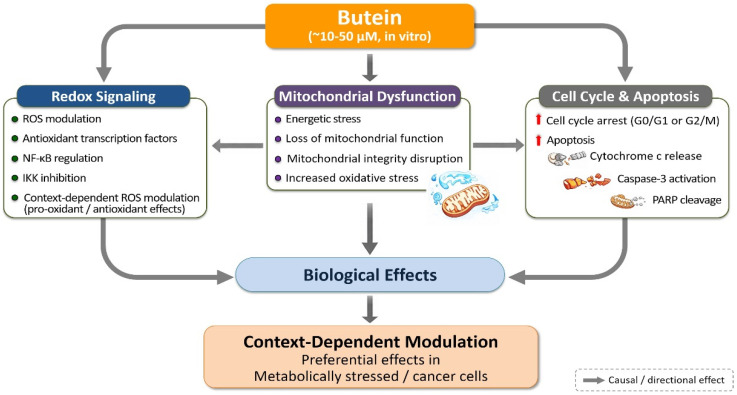
Integrated modulation of redox balance, mitochondrial function, and cell fate by butein. Butein exerts coordinated regulatory effects on redox-sensitive signaling, mitochondrial integrity, and apoptotic execution in experimental models. Through electrophile-driven interactions with redox-sensitive proteins, butein modulates inflammatory and pro-survival signaling pathways, including NF-κB activation, while inducing context-dependent changes in intracellular reactive oxygen species (ROS). Concurrently, butein perturbs mitochondrial membrane potential and oxidative phosphorylation, contributing to energetic stress and reduced ATP availability. These redox and mitochondrial perturbations may increase susceptibility to apoptotic signaling, and are associated with cell cycle arrest, caspase activation, and PARP cleavage. This schematic highlights a systems-level framework by which butein integrates redox regulation, metabolic disruption, and cell fate control under experimental conditions relevant to disease-associated cellular models. These mechanistic relationships are primarily supported by preclinical studies conducted at micromolar concentrations commonly used in vitro and reflect context-dependent modulation of interconnected redox and metabolic pathways.

The metabolic effects of butein are closely linked to its modulation of survival signaling pathways. Inhibition of Akt and ERK signaling restricts anabolic processes, mitochondrial biogenesis, and adaptive metabolic responses, thereby intensifying energetic stress in preclinical experimental systems [148,149,150]. Such coordinated effects are generally more pronounced at pharmacologically relevant concentrations, suggesting a dose-dependent amplification of metabolic vulnerability. In contrast to genistein, which predominantly affects glycolytic regulation, butein exerts a more pronounced influence on mitochondrial integrity and redox-dependent metabolic control. This distinction underscores the complementary metabolic regulatory profiles of these two polyphenols. However, the extent of these differential effects may depend on exposure level and cellular context, particularly when comparing pharmacological and physiological conditions. A comparative summary of the mitochondrial and metabolic effects of butein and genistein is provided in Table 6, and the integrated regulation of redox balance, mitochondrial function, and cell fate by butein is schematically illustrated in Figure 5.

### 4.3. Induction of Cell Cycle Arrest and Apoptotic Cell Fate by Butein

Butein has been widely reported to induce growth inhibition and apoptotic cell death across diverse experimental models, with its effects closely linked to cellular redox status, mitochondrial integrity, and stress adaptation in experimental systems [130,131,132,139,140,141]. Importantly, most of these effects have been characterized in vitro using micromolar concentrations of butein (typically ~10–50 μM), which may exceed physiologically achievable plasma or tissue levels, necessitating careful interpretation of their translational relevance. Similarly to genistein, the precise nature of cell cycle perturbation and apoptotic execution induced by butein varies depending on cell type, redox context, and metabolic state. Nevertheless, a recurring theme across studies is the ability of butein to shift cell fate decisions toward growth arrest and programmed cell death under conditions of pathological stress in preclinical experimental models [135,136,137,138].

At the level of cell cycle regulation, butein has been shown to interfere with cell cycle progression through modulation of cyclins, cyclin-dependent kinases, and checkpoint regulators, leading to cell cycle arrest at the G0/G1 or G2/M phases in susceptible cells [134,135,136,137]. These regulatory effects are most consistently observed at concentrations ≥ 10–20 μM, whereas lower concentrations (≤1–5 μM) may exert more modest or context-dependent influences on cell cycle progression in experimental observations. These effects are frequently associated with oxidative and metabolic stress signals rather than direct inhibition of canonical cell cycle machinery, highlighting the indirect yet effective control of proliferation exerted by butein through stress-responsive regulatory pathways [138,139].

In parallel, butein robustly activates apoptotic signaling, predominantly through the intrinsic mitochondrial pathway. Disruption of mitochondrial membrane potential, altered mitochondrial reactive oxygen species (ROS) production, and impaired oxidative phosphorylation collectively promote mitochondrial outer membrane permeabilization and the release of pro-apoptotic factors in cell-based models [145,146,147]. These apoptotic responses are strongly dose-dependent and are most prominently observed at concentrations ≥ 20–50 μM, suggesting that robust apoptosis induction may primarily reflect pharmacological exposure conditions. This sequence culminates in the activation of initiator and effector caspases and the cleavage of poly(ADP-ribose) polymerase (PARP), indicating irreversible commitment to apoptotic cell death in preclinical studies [147,148,149,150]. In certain cellular contexts, redox-mediated modulation of death receptor-associated signaling may further amplify apoptotic execution under experimental conditions [139,140,141].

Importantly, butein-induced apoptosis does not occur as an isolated event but emerges from the convergence of upstream regulatory perturbations. Redox imbalance and suppression of inflammatory signaling attenuate pro-survival cues, while mitochondrial dysfunction and energetic stress restrict the cellular capacity for adaptation in experimental systems [139,140,141,142,143,148,149,150]. At lower, physiologically achievable concentrations (≤1–5 μM), these effects may be attenuated or require prolonged exposure to achieve comparable outcomes, highlighting the importance of both dose and exposure duration. Together, these effects lower the apoptotic threshold and sensitize cells to programmed cell death. This coordinated cascade—redox disruption, mitochondrial stress, and apoptotic execution—is conceptually integrated in Figure 5.

From a systems biology perspective, butein functions as a regulator of cell fate rather than a nonspecific cytotoxic compound. By simultaneously modulating redox homeostasis, mitochondrial metabolism, and survival signaling, butein selectively promotes apoptosis in cells dependent on dysregulated stress-response and metabolic pathways in experimental disease models, while sparing non-malignant cells with intact adaptive capacity [130,131,132,144]. This apparent selectivity may, in part, reflect the differential sensitivity of transformed cells to redox and metabolic perturbations at higher butein concentrations, whereas normal cells exposed to lower physiological levels remain comparatively less affected. This multi-layered regulation reflects a defining characteristic of bioactive polyphenols and provides a mechanistic basis for the favorable selectivity observed for butein in experimental disease models.

Collectively, these findings support the positioning of butein as a mechanistically complementary partner to genistein in combination-based experimental frameworks. Whereas genistein primarily targets growth factor-associated signaling and glycolytic regulation, butein preferentially disrupts redox balance and mitochondrial function, with both compounds converging on apoptotic cell fate through distinct yet coordinated mechanisms [139,140,141,145,146,147,148,149,150]. However, the extent of this complementarity is likely influenced by exposure level and cellular context, particularly when comparing pharmacological and physiological conditions. This complementarity reinforces the rationale for integrative approaches aimed at coordinated modulation of survival, metabolic, and stress-response networks in complex disease contexts requiring further experimental validation.

## 5. Converging Pathways and Potential Synergistic Effects of Genistein and Butein

Despite the expanding literature on dietary polyphenols, translation of these compounds into clinically and nutritionally relevant applications remains challenging due to limitations related to bioavailability, metabolic transformation, and context-dependent biological activity [88,151,152,153,154]. Much of the currently available evidence derives primarily from in vitro experimental systems employing concentration ranges that may not fully reflect physiological exposure conditions in humans [155,156,157,158,159,160,161,162]. Therefore, mechanistic interpretations should be considered primarily as hypothesis-generating frameworks rather than direct indicators of clinical efficacy. Direct experimental studies specifically evaluating combined genistein–butein treatment remain limited, and the proposed interaction framework should therefore be interpreted as a concept-driven integration of complementary mechanistic evidence rather than as confirmation of pharmacological synergy.

Against this backdrop, genistein and butein—despite their substantial differences in chemical structure and primary molecular interactions—exert convergent effects on key regulatory pathways governing cell survival, metabolism, and cell fate decisions in experimental systems [98,99,100,163,164]. Experimental studies conducted predominantly in vitro suggest that both compounds can modulate PI3K/Akt and MAPK/ERK signaling pathways, which are central to the integration of growth factor stimulation with metabolic activity, stress adaptation, and resistance to apoptosis [165,166,167]. Persistent activation of these cascades is a hallmark of many cancers, contributing to metabolic reprogramming, uncontrolled proliferation, and therapeutic resistance [168,169]. However, many of these regulatory effects have been reported at micromolar concentrations that may exceed circulating levels achievable through dietary intake, and their physiological relevance remains to be clarified.

Beyond survival signaling, genistein and butein disrupt cellular metabolic homeostasis through partially distinct yet complementary mechanisms. Genistein primarily targets glycolytic regulation and glucose utilization, in part through suppression of Akt-dependent metabolic signaling and modulation of key enzymes such as hexokinase 2 and pyruvate dehydrogenase [170,171,172]. In contrast, butein exerts a more pronounced influence on mitochondrial function and redox balance, inducing mitochondrial dysfunction, impairing oxidative phosphorylation, and altering intracellular reactive oxygen species levels in preclinical models [117,141,173]. These observations are largely derived from controlled experimental models, and further investigation is required to determine whether similar regulatory effects occur under physiologically achievable exposure conditions.

The convergence of survival signaling inhibition and metabolic disruption creates a cellular environment conducive to apoptotic commitment in experimental contexts. Both genistein and butein activate caspase cascades and promote cleavage of poly(ADP-ribose) polymerase (PARP), indicating engagement of intrinsic apoptotic pathways [174,175,176,177]. Suppression of pro-survival signaling lowers the apoptotic threshold, while concomitant metabolic and redox stress further sensitizes cells to programmed cell death. Importantly, these findings should not be interpreted as evidence of pharmacological equivalence to established anticancer agents but rather as mechanistic indications that polyphenols may influence interconnected cellular regulatory systems.

From a mechanistic standpoint, the complementary targeting profiles of genistein and butein provide a strong biological rationale for their combined investigation. Whereas genistein predominantly modulates growth factor-associated signaling and glycolytic pathways, butein preferentially targets redox-sensitive signaling and mitochondrial integrity [178,179,180]. Simultaneous engagement of these regulatory layers may produce additive or context-dependent cooperative effects in experimental systems, although quantitative synergy and clinical relevance remain to be established through further experimental validation.

Combination-based strategies employing bioactive compounds have gained increasing attention as a means to explore coordinated modulation of complex regulatory networks. Such approaches emphasize multi-target, low-intensity regulatory interactions rather than single-pathway inhibition, consistent with systems-biology perspectives on network robustness and adaptive cellular responses [181,182,183]. However, the extent to which these mechanistic observations translate into measurable clinical benefit remains uncertain and requires further investigation in physiologically relevant models.

**Figure 6:**
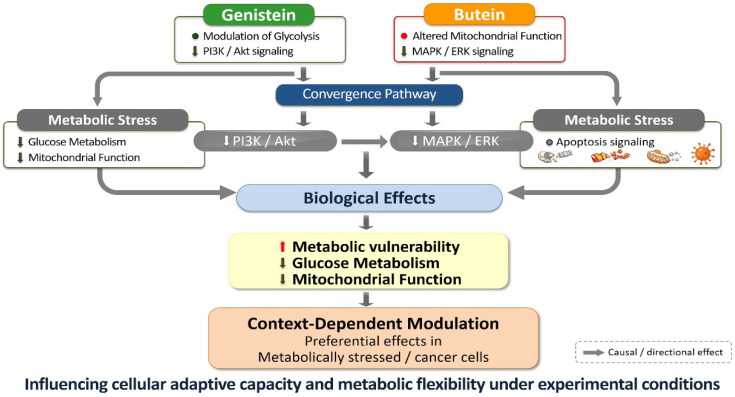
Convergent yet complementary regulatory actions of genistein and butein on survival signaling, metabolic homeostasis, and apoptotic commitment. Genistein primarily attenuates growth factor-associated signaling and glycolytic regulation through modulation of PI3K/Akt and MAPK/ERK pathways, thereby influencing proliferative signaling and metabolic flexibility in experimental systems. In contrast, butein preferentially targets redox-sensitive signaling and mitochondrial function, inducing oxidative and energetic stress through modulation of mitochondrial integrity and redox balance under experimental conditions. Coordinated engagement of these complementary regulatory layers may limit adaptive cellular responses and may increase susceptibility to apoptotic signaling through metabolic stress-associated sensitization. This systems-level framework illustrates how co-modulation of genistein and butein may contribute to the context-dependent modulation of interconnected cellular regulatory processes by simultaneously influencing survival signaling, metabolic reprogramming, and cell fate regulation under experimental conditions supported primarily by preclinical in vitro studies conducted at micromolar concentrations.

Collectively, the convergent yet non-redundant mechanisms of genistein and butein support their conceptual consideration as a mechanistically coherent bioactive combination targeting survival signaling, metabolic reprogramming, and apoptotic regulation in experimental research contexts. Further studies incorporating pharmacokinetic characterization, physiologically relevant exposure conditions, and in vivo validation will be necessary to clarify the magnitude and translational relevance of potential cooperative interactions between these compounds [47,184]. The complementary mechanisms underlying genistein–butein co-modulation are summarized in Table 7 and conceptually illustrated in Figure 6.

## 6. Therapeutic Implications in Cancer and Metabolic Diseases

### 6.1. Evidence from Preclinical Studies

A substantial body of preclinical evidence indicates that both genistein and butein influence disease-relevant signaling and metabolic pathways across a wide range of experimental models, including hormone-responsive tumors and metabolically active malignancies in experimental research contexts [185,186,187,188,189]. These effects have been primarily demonstrated in in vitro systems and selected in vivo models, where modulation of survival signaling, metabolic regulation, and apoptotic pathways has been observed [186,187,188]. However, much of this evidence derives from experimental conditions that may not fully reflect physiologically achievable plasma or tissue exposure levels in humans. Importantly, the reported biological activities of these compounds extend beyond simple growth inhibition and involve alterations in cellular metabolism, redox balance, and stress-response networks, which are increasingly recognized as determinants of disease-associated cellular vulnerability in preclinical studies [167,168,169,185].

Genistein has been widely reported to suppress tumor cell proliferation and enhance sensitivity to apoptotic stimuli through attenuation of PI3K/Akt and MAPK/ERK signaling, accompanied by coordinated disruption of glycolytic metabolism [185,186,187,188]. These findings have been observed predominantly in experimental model systems, and their magnitude may vary depending on dose, exposure context, and biological background. These effects are particularly evident in tumors characterized by aberrant growth factor signaling, increased glucose uptake, and dependence on glycolytic pathways for survival and proliferation in experimental models [98,99,100,165,166]. The interconnected effects of genistein on survival signaling, metabolism, and cell fate are conceptually integrated in Figure 4.

Similarly, butein has demonstrated the capacity to disrupt redox homeostasis and mitochondrial function, thereby inducing metabolic and oxidative stress that limits cellular adaptability and survival in experimental systems [189,190,191]. Preclinical studies show that butein induces mitochondrial dysfunction, alters intracellular reactive oxygen species levels, and suppresses redox-sensitive survival pathways, collectively lowering the threshold for apoptotic commitment. As with genistein, these observations are based largely on experimental systems, often at micromolar concentrations that may exceed physiologically achievable exposure conditions. These coordinated effects on redox balance, mitochondrial integrity, and apoptosis are schematically summarized in Figure 5, underscoring the systems-level nature of butein’s biological actions.

Although genistein and butein act through partially distinct molecular mechanisms, both converge on stress sensitization and apoptotic execution, suggesting a shared mechanistic framework rather than a confirmed therapeutic principle [117,141,172,173,174]. This convergence provides a strong mechanistic rationale for further investigation of combination-based experimental strategies. Preclinical investigations of such approaches suggest that simultaneous targeting of survival signaling, metabolic regulation, and redox balance may reduce compensatory mechanisms that often limit the efficacy of single-agent interventions in experimental models [192,193]. Nevertheless, the extent to which these coordinated effects represent true pharmacological synergy remains insufficiently defined. A comparative overview of these complementary mechanistic layers and their functional consequences is provided in Table 7.

It should be noted, however, that most available evidence remains preclinical. Direct demonstration of synergistic efficacy in clinically relevant models is still limited, highlighting the need for further investigation. In addition, the potential relevance of these findings to human disease should be interpreted cautiously, given the limited pharmacokinetic and tissue-distribution data currently available for both compounds. Future studies should incorporate quantitative combination analyses, dose–response modeling, and rigorously designed in vivo systems to better define the biological and translational relevance and context dependence of genistein–butein-based strategies [193,194].

### 6.2. Relevance to Nutritional and Preventive Medicine

Genistein and butein have attracted increasing attention in nutritional oncology and preventive medicine owing to their dietary origins and the long-term exposure of human populations to structurally related polyphenolic compounds [195,196,197]. Dietary polyphenols have been extensively studied as modulators of chronic disease risk, particularly in the contexts of inflammation, metabolic dysregulation, and cancer development [195,196]. Within this framework, genistein and butein exemplify bioactive compounds that may contribute to disease prevention through context-dependent modulation of biological pathways observed in experimental systems, rather than through acute cytotoxic effects [178,179,180]. However, the biological effects of isolated compounds observed in experimental systems should be distinguished from the complex physiological responses associated with whole-food consumption and dietary patterns.

Genistein, a major isoflavone present in soy-based diets, has been examined in epidemiological and experimental studies and has been associated with reduced incidence of certain hormone-related cancers and metabolic disorders [186,197]. These associations do not necessarily indicate direct causality and may reflect broader dietary, lifestyle, or metabolic factors. These observations are consistent with the mechanisms of growth factor signaling modulation and metabolic regulation discussed earlier and summarized in Table 5. Although butein is less prevalent in common diets, it is found in traditional medicinal plants and has gained interest as a nutraceutical due to its redox-modulating and anti-inflammatory properties [189,195]. Evidence supporting butein exposure in humans remains limited, and most mechanistic insights derive from experimental models conducted at concentration ranges that may exceed typical dietary intake levels. Its effects on redox balance, mitochondrial function, and apoptotic signaling, outlined in Table 6, suggest potential mechanistic relevance in preventive strategies targeting oxidative stress and metabolic imbalance.

The importance of genistein and butein in nutritional and preventive medicine does not lie in their use as standalone therapeutic agents but rather in their capacity to complement dietary patterns, lifestyle interventions, and conventional medical treatments [196,197,198]. From a systems biology perspective, prolonged exposure to low concentrations of bioactive polyphenols may contribute to gradual modulation of cellular regulatory networks involved in metabolic adaptation and stress responses under physiologically relevant exposure conditions. Nevertheless, the magnitude of these effects in humans remains uncertain, particularly given differences between experimentally applied concentrations and physiologically achievable exposure levels.

Translation of preclinical findings into nutritional or preventive applications faces several challenges, including limited oral bioavailability, interindividual variability in metabolism and gut microbiota composition, and uncertainty regarding effective dietary exposure levels [198,199,200]. In addition, complex interactions between dietary polyphenols and other nutritional components complicate extrapolation of experimental dosing paradigms to real-world settings. Accordingly, future research should integrate mechanistic studies with pharmacokinetic, nutritional, and formulation approaches to clarify the conditions under which genistein- and butein-based interventions may biologically meaningful regulatory effects under physiologically relevant exposure conditions [198,199,200,201].

**Figure 7:**
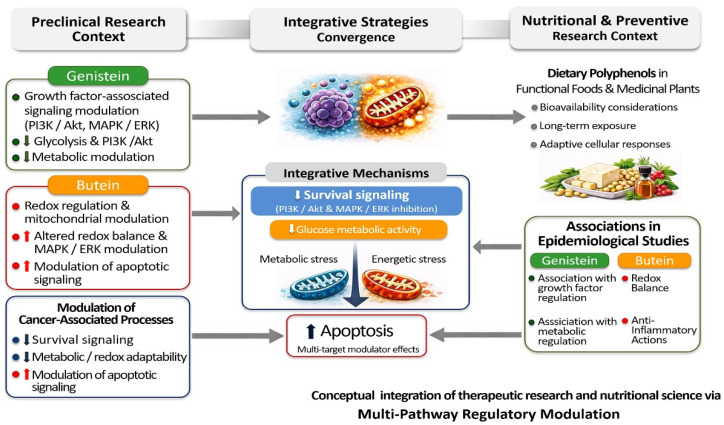
Conceptual positioning of genistein and butein across therapeutic and preventive contexts. This schematic illustrates the potential roles of genistein and butein in cancer research, metabolic disease-related cellular regulation, and preventive health contexts within experimental research frameworks. Through sustained, low-intensity modulation of survival signaling, metabolic regulation, redox balance, and apoptotic susceptibility, these bioactive polyphenols may contribute to context-dependent modulation of interconnected cellular regulatory networks without inducing substantial cytotoxicity under experimental conditions. In research-oriented therapeutic models, genistein and butein may be associated with increased stress sensitization and apoptotic responsiveness when examined in adjunct or combination-based experimental strategies. In preventive and nutritional contexts, long-term exposure to dietary or nutraceutical forms of these compounds may be associated with modulation of regulatory network behavior rather than selective targeting of isolated molecular components. These conceptual relationships are primarily supported by preclinical and mechanistic studies, and their potential biological relevance may depend on bioavailability, metabolism, and exposure conditions. This framework highlights systems-level modulation and context-dependent regulatory responses as defining features of genistein- and butein-associated approaches in experimental and hypothesis-generating contexts relevant to medicine and health research.

Overall, current evidence supports cautious consideration of these bioactive polyphenols as components of broader preventive and integrative strategies for cancer and metabolic diseases rather than as replacements for established therapeutic modalities [196,201]. Their proposed roles should therefore be interpreted within the context of dietary exposure, metabolic variability, and the current limitations of human pharmacokinetic data. Figure 7 conceptually summarizes the translational positioning of genistein and butein across therapeutic and preventive contexts.

## 7. Limitations and Future Perspectives

Another significant limitation is the predominance of in vitro data in the current literature. Although cell-based models have provided valuable mechanistic insights into the effects of genistein and butein on survival signaling, metabolic regulation, redox balance, and apoptotic pathways, the experimental evidence base remains uneven, with substantially fewer studies available for butein compared with genistein [202,203]. Moreover, existing animal studies often differ substantially in dosing regimens, treatment duration, formulation strategies, and experimental endpoints. Such heterogeneity complicates cross-study comparisons and limits the generalizability of reported outcomes [204]. Notably, there is currently a lack of clinical trials specifically designed to evaluate genistein–butein combination strategies, underscoring the early stage of translational research in this area and the need for systematic investigation under physiologically relevant conditions in appropriately designed experimental frameworks [205].

Looking forward, several research directions warrant focused and coordinated attention. First, optimization of dosing strategies and treatment schedules will be essential to determine whether the additive or context-dependent interactions suggested by mechanistic studies can be reproduced under biologically relevant exposure conditions in experimental and preclinical models [206]. This will require the application of quantitative combination analyses, dose–response modeling, and pharmacodynamic assessments to identify appropriate exposure ranges, biological contexts, and regulatory interactions. Such approaches are critical for distinguishing context-dependent modulation from simple additive effects in complex biological systems.

Advances in formulation and delivery technologies provide promising avenues to address bioavailability constraints. Nanoparticle-based carriers, lipid formulations, prodrug approaches, and food matrix-assisted delivery systems have demonstrated potential to improve the stability, absorption, and tissue distribution of polyphenols [94,207]. These strategies may facilitate improved systemic exposure while maintaining safety profiles consistent with experimental observations, although their clinical relevance remains to be established through further validation in physiologically relevant models.

Equally important is the development and application of more sophisticated in vivo and translational models. Studies employing metabolically relevant tumor models, patient-derived xenografts, or experimental systems that more accurately recapitulate the tumor microenvironment may yield deeper insights into how genistein and butein influence disease-relevant regulatory processes under controlled physiological conditions [208]. In parallel, integration of pharmacokinetic, metabolomic, and systems biology approaches could enhance understanding of how these compounds interact with endogenous signaling and metabolic networks, thereby improving mechanistic resolution and translational predictability [209].

Finally, future research should clearly distinguish between therapeutic and preventive research contexts. Higher exposure conditions used in experimental systems should not be directly interpreted as reflecting achievable dietary intake levels, and current evidence remains insufficient to define specific intake regimens or clinical dosing strategies [210]. Clarifying these distinctions will be essential for aligning experimental designs with intended clinical or public health objectives and for avoiding overinterpretation of pharmacological findings in nutritional contexts. Within this integrative framework linking mechanistic investigation and disease prevention research, progress will depend on bridging experimental insights with physiologically relevant models, thereby enabling the design of scientifically grounded translational studies.

## 8. Conclusions

Genistein and butein are structurally distinct yet mechanistically related bioactive polyphenols that modulate key signaling and metabolic pathways involved in disease-relevant cellular processes in experimental systems. Experimental evidence suggests that genistein, with more limited data available for butein, may modulate interconnected signaling and metabolic pathways associated with disease-relevant cellular processes under controlled experimental conditions [95,96,97,98,99,100,101,102,103,117,130,131,132,133,134,135,136,137,138,139,141,172,173,174]. These shared biological responses, arising from partially overlapping yet non-identical molecular interactions, highlight a systems-level mode of action that may be relevant to complex diseases such as cancer and metabolic disorders, where redundancy and adaptability within regulatory networks often limit the effectiveness of single-target approaches.

A central theme emerging from the literature is the complementary regulatory profiles of genistein and butein. Genistein predominantly interferes with growth factor–mediated signaling and glycolytic regulation, thereby influencing proliferative signaling and metabolic flexibility in experimental models. In contrast, butein exerts regulatory effects on redox balance and mitochondrial integrity, contributing to oxidative and energetic stress responses observed primarily in preclinical systems [108,109,110,111,112,113,114,115,116,117,118,138,139,140,141,142,143,144,145,146,147]. Convergence of these regulatory mechanisms at the levels of metabolic stress and apoptotic signaling provides a mechanistic rationale for further investigation of combination-based experimental approaches aimed at limiting compensatory signaling and metabolic adaptations that frequently reduce responsiveness to single-agent interventions [175,176,177,211,212]. Importantly, this rationale is grounded in mechanistic integration and pathway convergence rather than in assumptions of pharmacological synergy, underscoring the need for careful contextual interpretation.

Despite these mechanistic insights, several limitations constrain immediate translational interpretation. The majority of available evidence is derived from in vitro studies and selected animal models, and challenges related to bioavailability, interindividual metabolic variability, and physiologically achievable exposure levels remain significant considerations for translational relevance [202,203,204,213,214,215]. Moreover, direct experimental evidence supporting the efficacy of genistein–butein combination strategies in clinically relevant settings remains limited, highlighting the need for rigorously designed in vivo studies, quantitative combination analyses, and well-controlled translational investigations [205,208].

Beyond therapeutic research contexts, genistein and butein have also been investigated within nutritional and preventive research frameworks. However, interpretation of potential health relevance should consider that dietary exposure typically involves complex mixtures of bioactive compounds present in whole foods, making it difficult to attribute biological effects to a single phytochemical in isolation [195,196,197,198,199,200,201,210]. Accordingly, observed associations related to dietary patterns may reflect combined or context-dependent effects of multiple nutritional components rather than the action of an individual compound alone. Clear differentiation between experimental pharmacological conditions and dietary exposure contexts will therefore be essential for setting realistic expectations and guiding future research. Additional considerations relevant to translational interpretation include potential context-dependent estrogen receptor-related activity of genistein and electrophile-mediated protein interactions associated with butein, both of which may influence biological responses depending on exposure level, tissue environment, and metabolic state.

In summary, genistein and butein illustrate how structurally diverse bioactive compounds may converge on interconnected regulatory networks controlling cellular signaling, metabolism, and stress responses. Continued investigation of their molecular mechanisms, pharmacokinetic behavior, and context-dependent biological effects—particularly within integrative and systems-level frameworks—will be important for clarifying the biological contexts in which these polyphenols may provide mechanistic insight into disease-associated cellular regulation and support hypothesis-driven research in complex biological systems.

## Figures and Tables

**Table 2:** Structural features of genistein and functional relevance.

Structural Feature	Functional Implication	Key References
Isoflavone backbone	Planar scaffold; multi-target protein interactions observed in experimental models	[62,70]
4′,5,7-hydroxyl groups	Redox activity; hydrogen bonding with kinases reported in biochemical and cellular studies	[63,70]
17β-Estradiol similarity	Preferential ERβ binding; context-dependent biological responses primarily demonstrated in preclinical models	[64,65,66]
Aromatic ring system	π–π interactions with signaling proteins identified in structural and molecular studies	[67,68,69]
Molecular rigidity	Stabilization of ligand–protein interactions; modulation of metabolic and survival signaling pathways observed under experimental conditions	[59,60,61]
Active concentration range	Commonly studied in vitro concentrations: 10–50 μM; physiologically achievable concentrations in vivo: pico- to low nanomolar range	[66,70]

**Table 3:** Structural features of butein and functional relevance.

Structural Feature	Functional Implication	Key References
Chalcone backbone (open-chain)	Conformational flexibility; facilitation of non-receptor-mediated molecular interactions	[74,81]
α,β-unsaturated carbonyl	Michael acceptor; redox-sensitive proteins in biochemical and cellular studies	[75,76,77]
Multiple hydroxyl groups	Antioxidant capacity; hydrogen bonding with proteins involved in redox regulation	[78,79,80]
Electrophilic reactivity	Modulation of NF-κB, Akt, ERK signaling primarily observed in experimental models	[77,78,79]
Complementarity to genistein	Non-redundant targeting of redox and mitochondrial regulatory pathways under experimental conditions	[81,82]
Active concentration range	Commonly studied concentrations in vitro: >~50 μM; physiologically achievable concentrations in vivo remain insufficiently characterized but are considered likely to be substantially lower due to limited bioavailability	[81,82]

**Table 4:** Pharmacokinetic constraints of genistein and butein and translational implications.

Compound	Key Constraint	Mechanistic Basis	Physiological Context	Translational Opportunity
Genistein	Low aglycone exposure	Extensive phase II metabolism	Intestinal and hepatic exposure to conjugates (circulating levels: pico- to nanomolar; local exposure may be higher)	Metabolite-associated activity; local tissue exposure; potential relevance for combination-based experimental approaches
Butein	Short plasma half-life	Rapid metabolic clearance	Transient redox signaling modulation (primarily observed in vitro at micromolar concentrations)	Low-dose experimental activity; complementary mechanistic targeting
Both	In vitro–In vivo gap	Structure-driven metabolism	Tissue- and metabolism-dependent activity (micromolar in vitro vs. pico- to nanomolar in vivo)	Multi-target modulation at experimentally relevant exposure ranges
Both	Variable bioavailability	Microbiota and dietary matrix effects	Interindividual variability influenced by gut microbiota and dietary context	Dietary-context considerations; formulation-based experimental strategies

Note: This table summarizes conceptual limitation–opportunity relationships primarily derived from experimental evidence. Reported biological effects are frequently observed at micromolar concentrations in vitro, whereas physiologically achievable concentrations in vivo are typically in the pico- to nanomolar range, indicating a context-dependent gap that should be carefully considered when interpreting biological and translational relevance.

**Table 5:** Systems-level regulatory layers targeted by genistein (with representative in vitro concentration ranges observed in experimental models) and associated functional consequences observed in experimental models.

Regulatory Level	Primary Targets /Processes	Mode of Regulation by Genistein	Functional Consequence	Typical Concentration Range (In Vitro)
Survival signaling	PI3K/Akt pathway	Context-dependent attenuation of Akt phosphorylation	Reduced pro-survival signaling; decreased resistance to stress	≥10 μM
	MAPK/ERK pathway	Suppression of ERK activation and growth-related transcription	Inhibition of proliferative and adaptive responses	≥10 μM
Downstream survival effectors	mTOR, GSK-3β	Reduced downstream signaling activity	Impaired protein synthesis and cell cycle progression	≥10–20 μM
Metabolic regulation	Glucose uptake, glycolytic flux	Decreased glucose consumption and lactate production	Loss of metabolic flexibility	~10–50 μM
	Key metabolic enzymes (HK2, PDH)	Functional downregulation of glycolytic and mitochondrial entry points	Loss of metabolic flexibility	~10–50 μM
Energy-sensing pathways	AMPK signaling	Indirect activation via ATP depletion	Constraint of anabolic processes and cell growth	≥20–50 μM
Cell cycle control	Cyclins, CDKs, p21^Cip1^, p27^Kip1^	Checkpoint activation at G0/G1, S, or G2/M phases	Cell cycle arrest	≥10–20 μM
Apoptotic machinery	Mitochondrial membrane integrity	Induction of mitochondrial depolarization and cytochrome c release	Initiation of intrinsic apoptotic signaling	≥20–50 μM
	Caspase cascade, PARP	Caspase-3 activation and PARP cleavage	Irreversible apoptotic execution	≥20–50 μM
Systems-level outcome	Survival–metabolism–cell fate coupling	Coordinated multi-level modulation	Modulation of proliferation and apoptotic susceptibility under experimental conditions	Dose-dependent (≥10–50 μM)

**Table 6:** Systems-level regulatory layers targeted by butein (with representative in vitro concentration ranges) and functional consequences.

Regulatory Level	Primary Targets /Processes	Mode of Regulation by Butein	Functional Consequence	Typical Concentration Range (In Vitro)
Redox and inflammatory signaling	NF-κB pathway, redox-sensitive regulators	Electrophile-driven modulation of cysteine residues; suppression of NF-κB activation	Reduced inflammatory and pro-survival gene expression	≥10 μM
Survival signaling (redox-mediated, indirect)	PI3K/Akt, MAPK/ERK pathways	Redox-dependent attenuation of survival signaling	Sensitization to growth inhibition and apoptotic stimuli	≥10 μM
Mitochondrial function	Mitochondrial membrane potential, OXPHOS	Disruption of mitochondrial integrity and oxidative phosphorylation	Energetic stress and reduced ATP production	≥10–20 μM
Redox–metabolic coupling	Mitochondrial ROS production	Context-dependent modulation of ROS levels (pro-oxidant in stressed cells)	Altered redox balance and increased metabolic vulnerability	~10–50 μM
Metabolic regulation	Mitochondrial energy metabolism	Impaired bioenergetic efficiency and adaptive capacity	Reduced metabolic flexibility under stress conditions	≥10–20 μM
Cell cycle control	Cyclins, CDKs, checkpoint regulators	Stress-associated regulation leading to cell cycle arrest (G0/G1 or G2/M)	Restriction of proliferative capacity	≥10–20 μM
Apoptotic machinery	Mitochondrial pathway, caspase cascade	Induction of mitochondrial dysfunction, caspase-3 activation, and PARP cleavage	Irreversible apoptotic execution	≥20–50 μM
Systems-level outcome	Redox–mitochondria–cell fate coupling	Coordinated, context-dependent multi-level modulation	Modulation of growth and apoptotic susceptibility under experimental conditions	Dose-dependent (≥10–50 μM)

**Table 7:** Convergent and complementary mechanisms underlying the conceptual interaction framework of genistein and butein.

Regulatory Domain	Genistein	Butein	Convergent Functional Outcome
Primary molecular emphasis	Growth factor-associated signaling	Redox-sensitive signaling and mitochondrial regulation	Multi-layered modulation of survival-related regulatory networks (primarily observed in experimental models)
PI3K/Akt signaling	Direct attenuation of Akt phosphorylation and downstream metabolic signaling	Redox-mediated, indirect suppression of Akt activity	Reduced pro-survival signaling and increased susceptibility to stress responses under experimental conditions
MAPK/ERK signaling	Modulation of ERK activation and growth-promoting transcription	Redox-dependent interference on ERK signaling	Regulation of proliferation- and stress-associated signaling pathways (reported mainly in vitro)
Metabolic regulation	Modulation of glycolysis and glucose utilization (HK2, PDH-associated regulation)	Influence on mitochondrial function and oxidative phosphorylation	Induction of metabolic stress and altered bioenergetic balance in experimental systems
Redox balance	Indirect modulation via signaling–metabolism coupling	Context-dependent modulation of intracellular ROS dynamics	Potential sensitization to redox-associated cellular stress responses
Mitochondrial integrity	Secondary effects via metabolic perturbation	Modulation of mitochondrial membrane potential and function	Altered mitochondrial functional state associated with cellular stress adaptation
Apoptotic signaling	Caspase activation and PARP cleavage (reported in experimental models)	Caspase activation and PARP cleavage (reported in experimental models)	Engagement of apoptosis-related molecular markers under controlled experimental conditions
Adaptive capacity	Limitation of glycolytic adaptive responses	Limitation of mitochondrial and redox adaptive responses	Potential reduction in cellular adaptive flexibility in response to stress stimuli
Systems-level interaction	Signaling–glycolysis regulatory coupling	Redox–mitochondria regulatory coupling	Conceptual convergence across interconnected regulatory layers
Overall biological implication	Growth regulatory modulation and stress sensitization	Apoptosis-associated priming and metabolic perturbation	Additive or context-dependent cooperative interactions suggested in experimental models; quantitative synergy and physiological relevance require further validation

Note: This table summarizes mechanistic trends primarily derived from in vitro experimental studies. The described interactions reflect conceptual convergence across interconnected signaling and metabolic pathways and should not be interpreted as confirmation of clinical synergy or therapeutic efficacy. Further in vivo validation and pharmacokinetic characterization are required to determine physiological relevance.

## Data Availability

The original contributions presented in the study are included in the article; further inquiries should be directed to the corresponding author.

## Abbreviations

The following abbreviations are used in this manuscript:

Akt	Protein kinase B
AMPK	AMP-activated protein kinase
BTN	Butein
CDK	Cyclin-dependent kinase
ER	Estrogen receptor
ERα	Estrogen receptor alpha
ERβ	Estrogen receptor beta
ERK	Extracellular signal-regulated kinase
GEN	Genistein
GSK-3β	Glycogen synthase kinase-3 beta
HK2	Hexokinase 2
MAPK	Mitogen-activated protein kinase
mTOR	Mechanistic target of rapamycin
NF-κB	Nuclear factor kappa B
OXPHOS	Oxidative phosphorylation
PARP	Poly(ADP-ribose) polymerase
PDH	Pyruvate dehydrogenase
PI3K	Phosphoinositide 3-kinase
ROS	Reactive oxygen species
